# Characterization of rubber particles and rubber chain elongation in *Taraxacum koksaghyz*

**DOI:** 10.1186/1471-2091-11-11

**Published:** 2010-02-19

**Authors:** Thomas Schmidt, Malte Lenders, Andrea Hillebrand, Nicole van Deenen, Oliver Munt, Rudolf Reichelt, Wolfgang Eisenreich, Rainer Fischer, Dirk Prüfer, Christian Schulze Gronover

**Affiliations:** 1 Institut für Biochemie und Biotechnologie der Pflanzen, Westfälische Wilhelms-Universität Münster, Hindenburgplatz 55, 48143 Münster, Germany; 2 Institut für Medizinische Physik und Biophysik, Westfälische Wilhelms-Universität Münster, Robert-Koch Str. 31, D-48149 Münster, Germany; 3 Department Chemie, Lehrstuhl für Biochemie, Technische Universität München, Lichtenbergstrasse 4, 85748 Garching, Germany; 4 Fraunhofer Institut für Molekularbiologie und Angewandte Ökologie, Forckenbeckstr. 6, 52074 Aachen, Germany

## Abstract

**Background:**

Natural rubber is a biopolymer with exceptional qualities that cannot be completely replaced using synthetic alternatives. Although several key enzymes in the rubber biosynthetic pathway have been isolated, mainly from plants such as *Hevea brasiliensis*, *Ficus spec. *and the desert shrub *Parthenium argentatum*, there have been no *in planta *functional studies, e.g. by RNA interference, due to the absence of efficient and reproducible protocols for genetic engineering. In contrast, the Russian dandelion *Taraxacum koksaghyz*, which has long been considered as a potential alternative source of low-cost natural rubber, has a rapid life cycle and can be genetically transformed using a simple and reliable procedure. However, there is very little molecular data available for either the rubber polymer itself or its biosynthesis in *T. koksaghyz*.

**Results:**

We established a method for the purification of rubber particles - the active sites of rubber biosynthesis - from *T. koksaghyz *latex. Photon correlation spectroscopy and transmission electron microscopy revealed an average particle size of 320 nm, and ^13^C nuclear magnetic resonance (NMR) spectroscopy confirmed that isolated rubber particles contain poly(*cis*-1,4-isoprene) with a purity >95%. Size exclusion chromatography indicated that the weight average molecular mass (w) of *T. koksaghyz *natural rubber is 4,000-5,000 kDa. Rubber particles showed rubber transferase activity of 0.2 pmol min^-1 ^mg^-1^. *Ex vivo *rubber biosynthesis experiments resulted in a skewed unimodal distribution of [1-^14^C]isopentenyl pyrophosphate (IPP) incorporation at a w of 2,500 kDa. Characterization of recently isolated *cis*-prenyltransferases (CPTs) from *T. koksaghyz *revealed that these enzymes are associated with rubber particles and are able to produce long-chain polyprenols in yeast.

**Conclusions:**

*T. koksaghyz *rubber particles are similar to those described for *H. brasiliensis*. They contain very pure, high molecular mass poly(*cis*-1,4-isoprene) and the chain elongation process can be studied *ex vivo*. Because of their localization on rubber particles and their activity in yeast, we propose that the recently described *T. koksaghyz *CPTs are the major rubber chain elongating enzymes in this species. *T. koksaghyz *is amenable to genetic analysis and modification, and therefore could be used as a model species for the investigation and comparison of rubber biosynthesis.

## Background

Natural rubber poly(*cis*-1,4-isoprene) with a molecular mass of 10-10,000 kDa is one of the most important industrial raw materials in the world, and its sole commercial source is currently the para rubber tree *Hevea brasiliensis *[1]. Other sources, such as Russian dandelion (*Taraxacum koksaghyz *Rodin) and Guayule (*Parthenium argentatum*), could be useful in the event of supply shortages as well as providing a suitable alternative for people with allergies to hevein, a major allergen present in *H. brasiliensis *latex [2,3].

The biosynthesis of natural rubber takes place in the latex of laticifers or specialized parenchyma cells in the bark [1], where it is stored in rubber particles as an end product. Ultrastructural analysis of rubber particles from different species [4-6] revealed an almost identical globular structure that contains a homogeneous hydrophobic rubber core surrounded by an intact monolayer membrane. The monolayer membrane includes a mixture of lipids, proteins and other molecules with the hydrophilic portions of the phospholipids and glycosylated particle-bound proteins facing the cytoplasm [6-10]. The size of rubber particles ranges from 0.08-2 μm in *H. brasiliensis*, 0.2-6.5 μm in *Ficus *species and 1-2 μm in *P. argentatum *[6,11].

Natural rubber is synthesized by adding activated 2-methyl-1,3-butadiene (isopentenyl diphosphate, IPP) to the growing chain [12,13]. This reaction is catalyzed by specific long-chain *cis*-prenyltransferases (CPTs, EC 2.5.1.20), which are probably located on the surface of rubber particles. According to their function, CPTs are classified as short-, medium- or long-chain polymerizing enzymes and can be distinguished from *trans*-prenyltransferases (TPTs) by the presence of five conserved protein motifs [14]. They are found in bacteria [15], yeast [16], animals including humans [17] and plants [18-22].

Recently, two CPTs (RER2 and SRT1) were isolated from *Saccharomyces cerevisiae *and were shown to be responsible for the biosynthesis of dolichol, a long-chain polyprenol with a saturated alpha-isoprene unit, which serves as a glycosyl carrier for protein glycosylation in the endoplasmic reticulum [23]. The first plant CPT was identified in *Arabidopsis thaliana *(ACPT), and appears to be required for normal growth and development [24]. The latex of *H. brasiliensis *contains at least two CPTs, designated HRT1 and HRT2 (for *Hevea *rubber transferase). The addition of recombinant HRT2 to washed latex particles supplemented with radioactively-labeled IPP resulted in the significant production of a high-molecular-weight labeled rubber product, whereas recombinant HRT1 showed no significant activity [25]. *In vitro*, initiation of rubber biosynthesis by HRT requires intact particles, isopentenyl diphosphate (IPP), allylic diphosphates such as farnesyl diphosphate (FPP) and divalent metal cations (Mg^2+ ^or Mn^2+^) as a co-factor [26-28]. However, all attempts to purify a functional rubber transferase from rubber particles have failed, suggesting that the native enzyme needs additional factors for its activity [29].

In this study, we report the comprehensive analysis of rubber particles from *T. koksaghyz*. The rubber particles contained very pure poly(*cis*-1,4-isoprene) and retained their capacity to produce natural rubber *ex vivo*. Immunological analysis revealed that CPTs associated with these particles remain fully functional when expressed as recombinant proteins either in *Saccharomyces cerevisiae *or tobacco protoplasts.

## Results and Discussion

### Physical characterization of purified *T. koksaghyz *rubber particles

**Figure 1:**
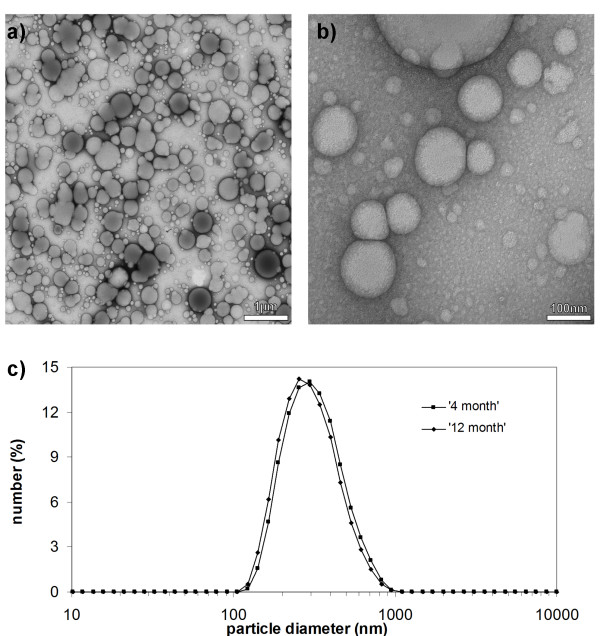
**Morphology of rubber particles from *Taraxacum koksaghyz***. Transmission electron micrographs at low a) and high b) magnification. c) Rubber particle size distribution measured by photon correlation spectroscopy in latex of 4- and 12-month-old plants. Magnifications in a) and b) are indicated by bar size.

Rubber particles examined by transmission electron microscopy at low magnification appeared approximately spherical, with diameters ranging from 0.2 to almost 1 μm (Figure 1a). At higher magnification, additional small and mostly spherical particles became visible with the smallest barely 10 nm in diameter (Figure 1b). Frequently, those rubber particles touching each other were deformed by the effect of surface tension in the staining solution during air-drying [30]. A similar unimodal particle size distribution ranging from 0.2-0.7 μm was observed by photon correlation spectroscopy, using rubber particles from the latex of 4-month-old and 1-year-old *T. koksaghyz *plants (Figure 1c). The average size of the rubber particles was 320 nm, and more than 50% of particles were in the size range 250-400 nm. The age of the plants appeared not to affect particle size. Size exclusion chromatography (SEC) analysis of both samples revealed an weight average molecular mass of ~ 4,750 kDa with a unimodal distribution, which again showed no significant variation as a function of plant age (data not shown).

### Concentration and chemical properties of *T. koksaghyz *rubber

**Figure 2:**
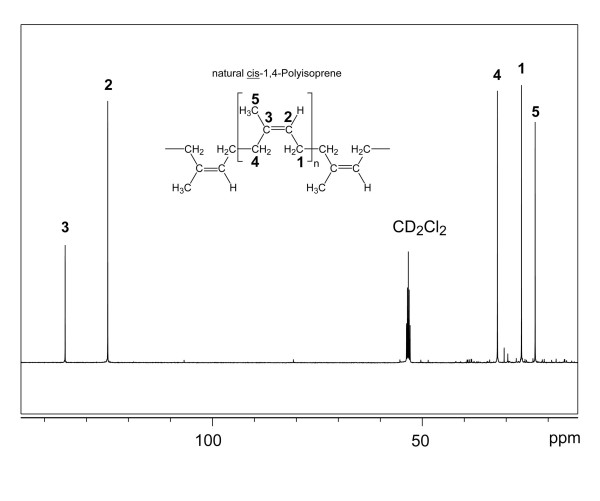
**^13^C NMR spectrum of rubber particles isolated from *Taraxacum koksaghyz***. The signal arising from the deuterated sample is indicated. The inset displays the structure and the carbon numbering for poly(*cis*-1,4-isoprene).

The concentration of rubber in the latex of greenhouse-cultivated *T. koksaghyz *plants was monitored from 4-18 months after sowing, revealing an increase during the first 8 months after which the concentration leveled off and remained constant at 130-150 mg dry rubber ml^-1 ^latex thereafter. Although the absolute rubber concentration varies in different *T. koksaghyz *accessions and under different growth conditions, our results agree with earlier investigations of rubber production in field-grown *T. koksaghyz *plants, where rubber accumulated only during the first growth season and then remained at constant levels [31]. To determine the chemical properties of *T. koksaghyz *rubber, we performed one- and two-dimensional NMR analysis of isolated rubber particles dissolved in dichloromethane. The ^13^C NMR spectrum displayed five dominant signals at chemical shifts indicative for poly(*cis*-1,4-isoprene), but not for poly(*trans*-1,4-isoprene) (Table 1 and Figure 2) [32]. Notably, the ^13^C NMR chemical shifts of C-4 and C-5 differ by more than 5 ppm for the *cis*- and *trans*-configured compounds (Table 1). Therefore, the observed frequencies leave no doubt that *T. koksaghyz *rubber is poly(*cis*-1,4-isoprene). Moreover, all ^1^H^13^C and ^1^H^1^H correlations detected in two-dimensional HMQC and COSY experiments, respectively, fully agreed with the couplings expected for poly(*cis*-1,4-isoprene) (Table 1). The NMR spectra contained few low-intensity signals arising from impurities, indicating that the purity of poly(*cis*-1,4-isoprene) in *T. koksaghyz *rubber is >95%.

**Table 1:** NMR data of poly(*cis*-1,4-isoprene) from *T. koksaghyz*.

	Chemical shifts*		Observed correlations in		**Published **^**13**^C-NMR chemical shifts**	
Position	^**1**^**H [ppm]**	^**13**^**C [ppm]**	COSY	HMBC	***cis*-Polyisoprene**	***trans*-Polyisoprene**
1	2.1	27.0	2	4	26.3	26.4
2	5.2	124.0	1, 5(w)***	5, 1, 4	124.8	123.9
3		134.2		1, 5	134.8	134.5
4	2.1	31.4		5, 1	31.8	39.3
5	1.7	22.3	1(w)	4	22.9	15.3

* referenced to the solvent signals; **[32]; *** (w), weak cross peak

The solvent was CD_2_Cl_2_.

### *In vitro *biosynthetic activity of rubber particles

The rubber biosynthetic activity of *T. koksaghyz *rubber particles was characterized *in vitro *by assaying the incorporation of the radiolabeled precursor IPP, which should become incorporated into the polymer and thus trapped in the rubber particles [21,33]. The IPP incorporation assay was carried out using intact isolated rubber particles as well as particles pre-treated with proteinase K to destroy particle-associated enzymes and other proteins. The treated and untreated particles were tested by SDS-PAGE and Coomassie Brilliant Blue staining, showing that the protein bands normally found in the particles were eliminated by proteinase K treatment (data not shown). This control allowed us to distinguish between physical and enzymatic incorporation of IPP into the particle, but had no influence either on particle integrity or stability. Earlier investigations into the *in vitro *activity of rubber particles used boiled particles as a control [33], which was not suitable for *T. koksaghyz *particles because of their rapid temperature-dependent agglomeration which changes the surface area to which IPP could be attached.

**Figure 3:**
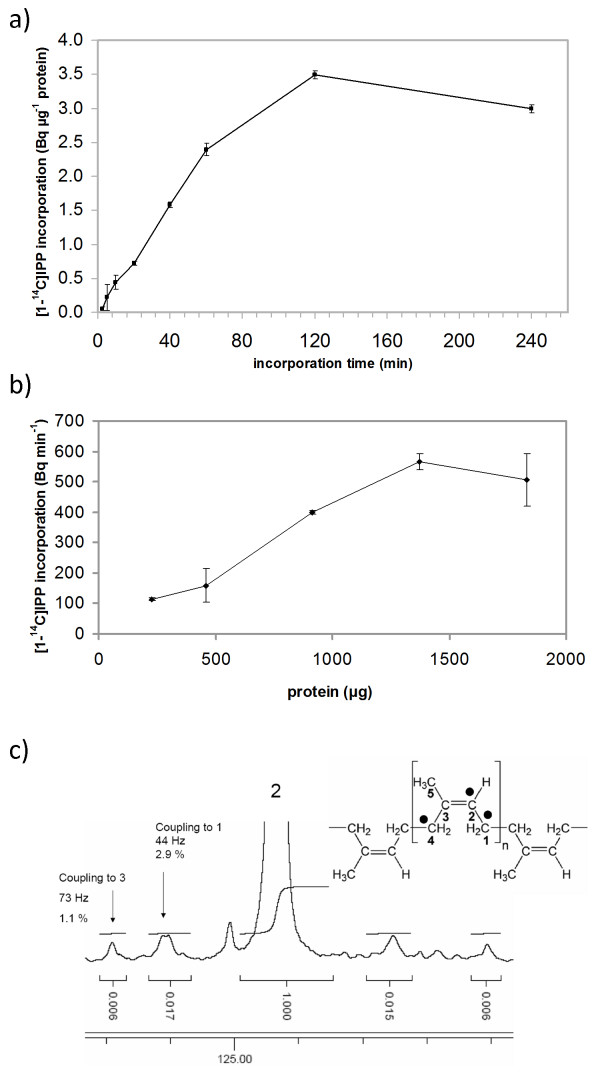
**Incorporation of IPP into rubber particles**. a) Time course-dependent and b) rubber particle protein dependent incorporation of [1-^14^C]IPP. c) ^13^C NMR signal of C-2 of poly(*cis*-1,4-isoprene) from the labeling experiment with [1,2,4-^13^C_3_]IPP. The filled circles indicate ^13^C atoms from [1, 2, 4-^13^C_3_]IPP. Values in a) and b) represent mean (± standard deviation) from three biological repetitions.

In time course experiments, *T. koksaghyz *rubber particles enzymatically incorporated increasing amounts of [1-^14^C]IPP over the incubation time (Figure 3a). Saturation was achieved after 2 h, possibly reflecting the loss of chain elongation activity due to the particles becoming unstable as reported for other rubber-producing plants [29]. The incorporation of [1-^14^C]IPP also increased as more rubber particles were added to the reaction, giving a sigmoid curve progression that reflected an enzymatic incorporation process. The enzymatic activity of *T. koksaghyz *rubber particles *in vitro *was about 0.2 pmol min^-1 ^mg^-1^.

To calculate the rubber transferase activity of the rubber particles, IPP incorporation was measured as a function of protein concentration (Figure 3b), whereas the mean amount of protein in *T. koksaghyz *rubber particles remained approximately constant at 70-80 μg mg^-1^. This allowed us to calculate the enzymatic [1-^14^C]IPP incorporation activity of 2.6-3 pmol min^-1 ^mg^-1 ^rubber particle protein, which corresponds to the findings for rubber particles from other plants (0.08-13 pmol min^-1 ^mg^-1 ^for *H. brasiliensis*, 0.03-6 pmol min^-1 ^mg^-1 ^for *F. carica *and 1.3 pmol min^-1 ^mg^-1 ^for *P. argentatum *[27,33,34]). The range of rubber particle activity observed within each species could reflect the different assay conditions and particle extraction methods. For example, rubber transferase activity is thought to be Mg^2+^-dependent [35], and the concentration of Mg^2+ ^may differ between species, whereas optimal concentrations for *H. brasiliensis *and *P. argentatum *were 2 mM and 8 mM, respectively [33,36]. We therefore varied the Mg^2+ ^concentrations in the *in vitro *rubber transferase assay for *T. koksaghyz *rubber particles, and found the optimal activity at 4-5 mM Mg^2+ ^(Table 2). To verify that rubber chain elongation in *T. koksaghyz *rubber particles occurred by the transfer of IPP in the *cis-*configuration, a sophisticated labeling assay was developed in which rubber particles were incubated with [1, 2, 4-^13^C_3_]IPP, vacuum dried and the residual material dissolved in CD_2_Cl_2 _for ^13^C NMR analysis. The ^13^C NMR signal of C-2 poly(*cis*-1,4-isoprene) (Figure 3c) showed, in addition to the intense central signal, pairs of coupling satellites characterized by coupling constants of 73 Hz (coupling to ^13^C-3) and 44 Hz (coupling to ^13^C-1). The C-1 satellite pair was approximately three times more intense than the C-3 pair (Figure 3c), indicating that isotopologues with adjacent ^13^C-1 and ^13^C-2 were slightly enriched in accordance with their biosynthetic origin from [1, 2, 4-^13^C_3_]IPP (cf. filled circles in Figure 3c). The low enrichment factor can be explained by the large excess of unlabeled poly(*cis*-1,4-isoprene) at the beginning of the labeling reaction. Nevertheless, the non-stochastic distribution of the satellite pairs provides additional evidence for *cis-*prenyltransferase activity under these experimental conditions.

**Table 2:** Effect of Mg^2+ ^ion on [1-^14^C]IPP incorporation of *T. koksaghyz *rubber particles.

**MgCl**_**2**_** [mM]**	**[1-**^**14**^**C]IPP incorporation** **[Bq μg**^**-1**^**protein]**
2 mM	0.57 (± 0.023)
3 mM	0.83 (± 0.037)
4 mM	0.89 (± 0.039)
5 mM	0.91 (± 0.040)
7.5 mM	0.52 (± 0.023)
10 mM	0.04 (± 0.002)

For the *ex vivo *rubber biosynthesis experiment 3 mg of freshly prepared rubber particles was incubated for 60 min with [1-^14^C]IPP in IPP assay buffer containing different concentrations of Mg^2+ ^ion. Values represent mean (± standard deviation) from three biological repetitions.

**Figure 4:**
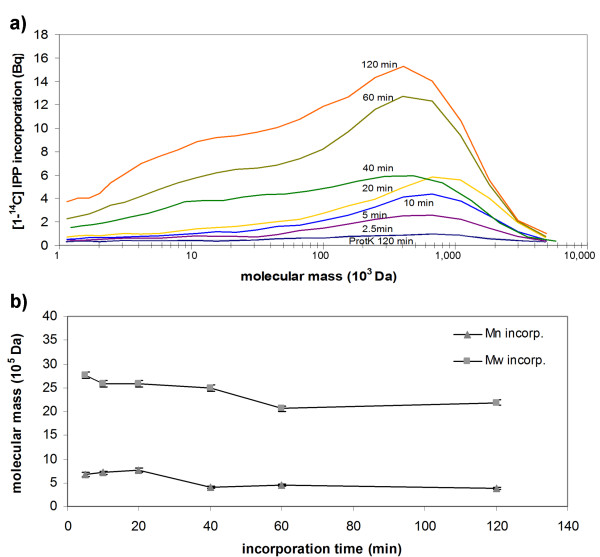
**Molecular mass distribution of [1-^14^C]IPP-labeled rubber synthesized *in vitro***. a) SEC profile of labeled material. b) w and n of the labeled polymer. IPP incorporation assay was performed and stopped after the time points shown. Extracted polymer material was fractionated by SEC and the radioactivity of the resulting fractions was determined by scintillation. Proteinase K (ProtK)-treated particles controlled for IPP trapped on the rubber particles through non-enzymatic mechanisms. Progression lines represent the mean of two measurements.

The distribution of [1-^14^C]IPP transfer to existing rubber molecules of different molecular masses was investigated by SEC (Figure 4 and Table 3). This revealed a typical skewed unimodal mass, with a weight average molecular mass (w) of 5,170 kDa (± 134 kDa) and number average molecular mass (n) of 2,460 kDa (± 142 kDa), indicating a low level polydispersity of 2.1. Again, the incorporation of [1-^14^C]IPP increased with the incubation time (Figure 4a). After 5 min the incorporation of [1-^14^C]IPP occurred at a significantly higher rate in untreated particles compared to those treated with proteinase K, whereas the means of labeled material were 2,760 kDa for w and 672 kDa for n, respectively. Furthermore, the SEC data indicated that *in vitro *rubber chain elongation occurred over a broad range (1-5000 kDa), whereas the highest incorporation rate was observed for molecules with w of 2,000-2,500 kDa. Interestingly, the w and n of the [1-^14^C]IPP labeled rubber decreased during the assay (Figure 4b), increasing the polydispersity (Table 3) and indicating both the preferential transfer of [1-^14^C]IPP to long-chain molecules and the possibility that new rubber molecules are synthesized after the depletion of the long-chain starter molecules. The lower molecular mass distribution of labeled molecules in comparison to the starting material suggested that chain termination by transferase complex might occur preferentially in molecules >2,500 kDa. These data indicate that *in vivo *rubber biosynthesis can be only partially reconstituted *in vitro *as also suggested by Tangpakdee *et al. *[37].

**Table 3:** Molecular mass and polydispersity of labeled and unlabeled material from *ex vivo *IPP incorporation.

rubber sample	**w (10**^**5**^** Da)**	**n (10**^**5**^** Da)**	**w n**^**-1**^
unlabeled _5-120 min_	51.7 (± 1.34)	24.6 (± 1.42)	2.1
labeled _5-20 min_	26.5 (± 1.01)	7.1 (± 0.45)	3.7
labeled _40-120 min_	22.5 (± 2.27)	4.1 (± 0.30)	5.5

In an *ex vivo *rubber biosynthesis experiment 6.5 mg of freshly prepared rubber particles was incubated with [1-^14^C]IPP for the time periods indicated. Extracted rubber material for each assay was fractionated by size exclusion chromatography and radioactivity levels were determined by scintillation.

### Particle associated *cis*-1,4-polyprenylcistransferases

**Figure 5:**
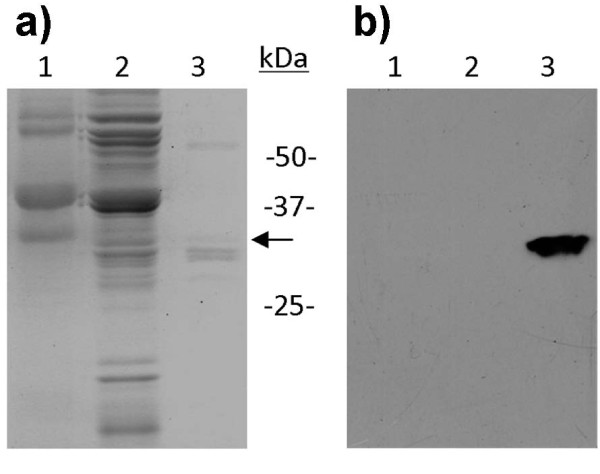
**Detection of *T. koksaghyz *CPT in the rubber phase**. Latex was harvested and divided into pellet (lane 1), C-serum (lane 2) and rubber phase (lane 3). Proteins were separated by SDS-PAGE and either stained with Coomassie Brilliant Blue a) or transferred to a membrane for western blot analysis using antibodies against TkCPTs b).

Recently, we identified and isolated three *T. koksaghyz *CPTs designated TkCPT1-3 [38]. The *TkCPT1 *and *TkCPT2 *cDNAs are each 927 bp in length and the *TkCPT3 *cDNA is 903 bp in length, giving proteins with molecular masses of 32-34 kDa. All three genes are strongly expressed in the latex, but only minimally expressed in other tissues such as leaves, pedicels and roots. Antibodies recognizing the three TkCPTs revealed a strong signal in western blots of the rubber particle fraction of *T. koksaghyz *latex, with an apparent molecular mass of 34 kDa (Figure 5). Although it has previously been suggested that rubber transferases are associated with rubber particles [39-41], our results for TkCPTs are the first to confirm this theory explicitly.

### Functional analysis of TkCPT1-3

**Figure 6:**
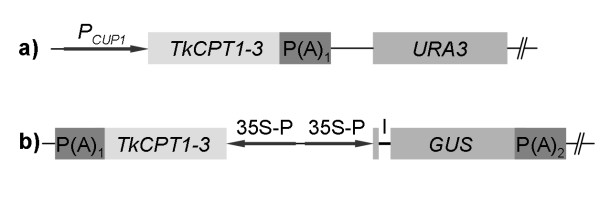
**Plasmids for heterologous expression of *TkCPT1-*3**. For heterologous expression of *TkCPT1-3 *in *S. cerevisiae *and protoplasts of *N. tabacum *the corresponding cDNAs were cloned into the plasmids pYEX-BX a) and pCAMBIA-1305.1 b), respectively. *PCUP1*, *S. cerevisiae CUP1 *promoter; P(A)_1_, cauliflower mosaic virus (CaMV) 35S polyadenylation signal sequence; *URA3*, *S. cerevisiae *URA3 locus; 35S-P, CaMV 35S promoter; *GUS*, beta-glucuronidase synthetic construct including catalase intron (I) (GenBank: AAK29426); P(A)_2_, *Agrobacterium tumefaciens *D-nopaline synthase polyadenylation signal sequence.

**Figure 7:**
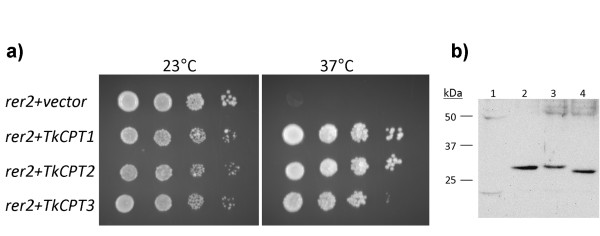
**Functional complementation of the temperature-sensitive dedol-PP synthase rer2 yeast mutant SNH23-D7 by TkCPTs**. a) Yeast strain SNH23-D7 transformed with plasmid pYEX-BX, pYEX-TkCPT1, pYEX-TkCPT2 and pYEX-TkCPT3 were dropped on SD-URA plates and incubated for 2 days at the temperatures shown. b) Western blots were performed with antibodies against TkCPTs to detect TkCPT in yeast SNH23-D7 (rer2) transformants pYEX-BX (vector control) (1), pYEX-TkCPT1 (2), pYEX-TkCPT2 (3) and pYEX-TkCPT3 (4).

To determine whether the three isolated CPT genes encode functional proteins with long chain *cis*-prenyltransferase activity *in vivo*, the cDNAs were expressed in the yeast strain SNH23-7D (Figure 6a), which is a temperature sensitive mutant for the dehydrodolichyl diphosphate (dedol-PP) synthase *rer2 *[16]. All three *T. koksaghyz *CPTs were able to suppress the growth deficiency phenotype of the *rer2 *mutant (Figure 7a), and western blot analysis of the complementing yeast transformants revealed the expression of full length TkCPT1-3 proteins (Figure 7b). The suppression of the temperature sensitive phenotype demonstrated clearly that TkCPT1-3 catalyze the synthesis of the *cis*-polyprenol dedol-PP, which has a molecular mass of 1.1-1.3 kDa. Similar results were obtained for HRT2, which is believed to be the long-chain rubber producing CPT in *H. brasiliensis *[25]. The formation of high-molecular-mass polyprenols in yeast is possibly repressed by the rapid de-phosphorylation of the dedol-PP to dolichol, which is not a substrate for the chain elongation process. It has previously been suggested that rubber transferases need co-factors for stability and activity [25,29] but our experiments indicate that, at least for *T. koksaghyz *CPTs under our experimental conditions, this does not appear to be the case.

More detailed analysis of TkCPT activity was carried out using *N. tabacum *mesophyll protoplasts transfected with each of the cDNAs (Figure 6b, Table 4). In order to avoid qualitative or quantitative differences in protoplast and/or plasmid DNA preparations interfering with the direct comparison of CPT activities between transfection experiments, beta-glucuronidase was co-expressed from the same plasmid as an internal standard as described by Lepetit *et al. *[42]. Protein extracts from protoplasts expressing each of the TkCPTs showed strong IPP transferase activity, with no significant difference between the three cDNAs. It is therefore most likely that all three enzymes act cooperatively in *T. koksaghyz *rubber biosynthesis.

**Table 4:** Heterologous expression of TkCPTs in *Nicotiana tabacum *protoplasts.

Enzymes	**GUS activity (*μ*mol min**^**-1 **^***μ*g**^**-1**^)	**CPT activity (pmol min**^**-1 **^**mg**^**-1**^)	CPT/GUS ratio
GUS/-	1.78 (± 0.10)	-	-
GUS/TkCPT1	1.34 (± 0.16)	3.48 (± 0.38)	2.73 (± 0.50)
GUS/TkCPT2	0.97 (± 0.15)	2.05 (± 0.37)	2.28 (± 0.70)
GUS/TkCPT3	1.22 (± 0.21)	2.90 (± 0.64)	2.50 (± 0.16)

Protoplasts were transfected with plasmids containing both *GUS *and *TkCPTs *or *GUS *alone in all cases controlled by the CaMV 35S promoter. Protoplast protein extracts were used for measurement of enzyme activities, whereas GUS activity served as the internal standard for normalization of transfection experiments. Values represent mean (± standard deviation) from six biological repetitions. No significant differences existed in enzyme activity between TkCPT1, TkCPT2 and TkCPT3, as calculated using Student's t-test (P, 0.01).

## Conclusion

We have investigated the physical properties and developmental profile of rubber particles from *T. koksaghyz *latex and have shown that they possess intrinsic *cis*-1,4-polyprenylcistransferase (rubber transferase) activity of 0.2 pmol min^-1 ^mg^-1 ^which can be partially reconstituted *in vitro *without further co-factors. We are the first to demonstrate conclusively that CPTs are an intrinsic part of the rubber particle and we have demonstrated a correspondence between the CPT activity in isolated rubber particles and the recently identified CPT genes *TkCPT1-3 *in *T. koksaghyz*, which are able to complement a yeast strain deficient in CPT activity and maintain their activity in tobacco protoplasts. *In vitro *rubber biosynthesis experiments resulted in a skewed unimodal distribution of [1-^14^C]isopentenyl pyrophosphate (IPP) incorporation at a weight average molecular mass of 2,500 kDa. Our data indicate that TkCPT1-3 are responsible for the rubber chain elongation that occurs in *T. koksaghyz *rubber particles and that their roles in this regard may be redundant. Our experiments provide crucial background information that will allow the development of *T. koksaghyz *as a potential alternative commercial source of rubber.

## Methods

### Plant material and cultivation conditions

*Taraxacum koksaghyz *plants were obtained from the Botanical Gardens Karlsruhe (Karlsruhe, Germany) and cultivated at 18°C with a 16-h photoperiod (20 klx) in controlled growth chambers or in the greenhouse. Plants were cultivated in a prefertilized 1:1 mixture of standard soil (ED73 Einheitserde, Fröndenberg, Germany) and garden mold (Botanical Garden Münster, Germany) and fertilized every 4 weeks with a commercial fertilizer according to the manufacturer's recommendations (Hakaphos Plus, Compo GmbH, Münster, Germany).

### Rubber preparation and determination

For the isolation of native and functional rubber particles we followed the general procedure described for *H. brasiliensis *[27,43] with the following modifications. Latex was harvested from petioles or roots of 20 week old *T. koksaghyz *plants, if not stated otherwise, by dissecting the tissue with a razor blade and transferring the expelling latex into an equal volume of ice-cold rubber extraction buffer (100 mM Tris.Cl (pH 7.8), 350 mM sorbitol, 10 mM NaCl, 5 mM MgCl_2_, 5 mM DTT) and centrifuging (12,000 × *g*, 20 min, 4°C). The latex separated into three fractions (pellet, C-serum and rubber phase) the latter two of which were transferred to a new tube and centrifuged as above. The rubber phase containing the rubber particles was transferred to a fresh tube and washed with 800 μl rubber extraction buffer and then dissolved in rubber extraction buffer and stored briefly at 4°C. To determine the dry rubber content, 20 μl of latex was transferred to a fresh tube and gently overlaid with 20 μl glacial acetic acid to coagulate the rubber particles. Afterwards, the coagulum was air-dried for 24 h.

### Photon correlation spectroscopy

Freshly prepared rubber particles were dispersed by brief ultrasonication and then filtered with a 5 μm syringe filter prior to dynamic light scattering with a Zetasizer Nano ZS (Malvern Instruments GmbH, Herrenberg, Germany) containing a He-Ne laser (4.0 mW at 633 nm) and an Avalanche photodiode detector with a Q.E. >50% at 633 nm. Measurements were performed according to the manufacturer's instructions.

### Transmission electron microscopy

Freshly prepared *T. koksaghyz *latex rubber phase was diluted 1:3 with rubber extraction buffer containing 1 mM DTT, and a small droplet was placed for 1 min on a freshly glow-discharged carbon layer (thickness ~ 10 nm) onto a Pioloform film supported by a commercial Cu-mesh grid. After removing excess liquid, the grid was washed with double-distilled water and stained with 2% aqueous uranyl acetate for 30 s before air drying. Transmission electron microscopy (TEM) was carried out using a Philips EM 410 (acceleration voltage 80 kV) in the bright-field mode, and micrographs were recorded on Imaging Plates (Ditabis, Pforzheim, Germany).

### IPP-incorporation assay

To measure the incorporation of IPP into rubber, the latex rubber phase was mixed with assay buffer to a final concentration of 100 mM Tris-HCl (pH 7.5), 2.5 mM CaCl_2_, 10 mM DTT, 1 mM sodium azide, 0.05% Triton X-100, 5 mM MgCl_2_, 2.8 μM E,E- farnesyl diphosphate (Sigma-Aldrich) and 7.2 μM [1-^14^C]- or [1, 2, 4-^13^C_3_]isopentenyl pyrophosphate (IPP) (GE Healthcare). The reaction mixture was incubated at 30°C for 2 h and then stopped by heating to 95°C for 5 min. The reaction products were hydrolyzed to corresponding alcohols using 2 μl of potato acid phosphatase (1 U μl^-1 ^dissolved in double-distilled water) and 398 μl phosphatase buffer (50 mM sodium acetate (pH 4.7), 0.1% Triton X-100, 60% (v/v) methanol) for 2 h at 37°C as described [44]. The products were extracted by shaking with 600 μl n-hexane for 1 h, air dried and resuspended in 300 μl n-hexane. Radioactivity was measured using a scintillation counter (Beckman Scintillation Counter LS6500) after mixing 50 μl of the extracts with 4 ml Rotiszint^® ^eco plus (Roth, Karlsruhe, Germany). Extracts were air dried and resolved in tetrahydrofuran. Size-exclusion chromatography to determine the molecular size distribution of natural rubber and rubber produced in radioactive assays was carried out as previously described [45].

### NMR spectroscopy

One-dimensional ^1^H and ^13^C NMR spectra were measured at 500 and 125 MHz, respectively, using a DRX500 or AVANCE 500 spectrometer (Bruker, Rheinstetten, Germany). Two-dimensional HMQC, HMBC and COSY spectra were measured with the AVANCE 500 spectrometer using an inverse probe-head and standard parameter sets implemented in TOPSPIN 1.1. The solvent was deuterated dichloromethane and the temperature was 27°C. The experimental time for ^13^C NMR spectra was typically 15 h (corresponding to more than 10,000 scans). Data were processed using TOPSPIN 1.1 or MestReNova.

### Construction of expression vectors for heterologous expression

RNA from *T. koksaghyz *latex was isolated as described previously [45] and cDNA synthesis was carried out using the SuperScript II™ Reverse Transcriptase Kit (Invitrogen, Karlsruhe, Germany) with an oligo(dT) primer. For all three *TkCPT*s (*TkCPT1*, *TkCPT2 *and *TkCPT3*) cDNA was generated using the primer combination cpt-TK_EcoRI (5'-AAA GAA TTC ATG CAA GTG AAT CCA ATC ATT ACT AC-3') and cpt-TK-rev_SalI (5'-AAA GTC GAC TTA TGC CTG CTT CTT CTT CTT CTC C-3'). The products were inserted into the pCRII-TOPO^® ^vector (Invitrogen, Karlsruhe, Germany), sequenced and then transferred using *Eco*RI and *Sal*I restriction sites into the expression vector pGEX4-T1 (GE Healthcare Europe GmbH, Freiburg, Germany). This allowed the expression of TkCPTs as N-terminal fusions to glutathione-S-transferase (GST) for heterologous expression in *Escherichia coli *BL21-cells and downstream antibody generation.

For heterologous expression of *TkCPT1-3 *in *Nicotiana tabacum *var. SR1 protoplasts, the corresponding cDNAs were amplified with the primers TkCPT_PciI (5'-AAA ACA TGT TAC AAG TGA ATC CAA TCA TTA CTA C-3') and TkCPT-rev_XbaI (5'-AAA ACA TGT TAC AAG TGA ATC CAA TCA TTA CTA C-3') and transferred using the *Nco*I and *Xba*I sites into the pUC18-based pAM vector containing the CaMV 35S promoter and polyadenylation sequences from pRT104 [46]. The cassette was then transferred using the *Kpn*I and *Eco*RI sites into pCAMBIA-1305.1 (GenBank: AF354045), which already contains the *gus*A reporter gene including a catalase intron under the control of the CaMV 35S promoter.

For expression in yeast, the *TkCPT *cDNAs were released from pAM using *Xho*I and *Eco*RI and introduced into vector pYEXBX (Clontech Laboratories Inc., Saint-Germain-en-Laye, France), which had been digested with *Sal*I and *Eco*RI.

### Generation of antibodies against TkCPTs

The three *TkCPT *clones in pGEX4-T1 were overexpressed in 300-ml cultures of *E. coli *strain BL21, induced with 1 mM isopropyl-beta-D-thiogalactopyranoside (IPTG). Purified TkCPT1 was sequenced by MALDI-MS and administered to rabbits by EUROGENTEC (Seraing, Belgium). The pre-immune and antibody sera were tested for specificity by western blot against the recombinant proteins TkCPT1, TkCPT2 and TKCPT3.

### SDS-PAGE and western blots

SDS-PAGE was carried out using 15 μg protein per lane from the pellet, C-serum and rubber phases of fresh *T. koksaghyz *latex. Protein concentrations were determined using the Bradford method [47]. Proteins were separated on SDS-PAGE gels and either stained with Coomassie Brilliant Blue or transferred to nitrocellulose membranes as described [48]. The membranes were incubated with the primary antibody (1:500 dilution) for 1 h at room temperature, washed and then incubated with a mouse anti-rabbit IgG conjugated to horseradish peroxidase (Sigma, Munich, Germany) according to the manufacturer's instructions. Membranes with HRP-coupled secondary antibodies were imaged on X-ray films by chemiluminescence detection.

### Expression of TkCPTs in yeast

Yeast strain SNH23-D7 (*MATa rer2-2 mfa1::ADE2 mfa2::TRP1 bar1::HIS3 ade2 trp1 his3 leu2 ura3 lys2*) [16] was cultivated in standard YPD medium for 48 h at 20°C, then transformed [49] with pYEXBX-*TkCPT1-3*, and the pYEXBX base vector as a control. After regeneration, cells were plated on SD-URA agar, colonies for each construct were transferred to 0.9% NaCl and adjusted to different optical densities (1, 0.1, 0.01 and 0.001) and 5-*μ*l droplets were spotted onto SD-URA plates. After 48 h incubation in SD-URA liquid medium, denatured protein extracts were prepared from 20 OD_600 _units [50] and used for SDS-PAGE and western blot analysis.

### Expression in protoplasts and GUS assay

Protoplasts were isolated from *Nicotiana tabacum *var. SR1 and Ca(NO_3_)_2 _polyethylene glycol-mediated DNA transfer was performed as described [51] using 3.3 × 10^5 ^protoplasts and 10 *μ*g of pCambia1305.1-*TkCPT1-3 *DNA per transfection. Frozen protoplasts were sheared in IPP assay buffer, briefly centrifuged and the supernatants pooled from six replicate transfections. Protein concentration was determined using the Bradford method [47] and bovine serum albumin as a standard. Approximately 100 μg of protein extract was used in the IPP incorporation assay and 1 μg in the glucuronidase activity assay with 4-methylumbelliferylglucuronide (4-MUG) as the substrate [52].

## Abbreviations

4-MUG: 4-methylumbelliferylglucuronide; ACPT: *Arabidopsis thaliana cis*-prenyltransferase; COSY: correlation spectroscopy; CPT: *cis*-1,4-polyprenylcistransferases; FPP: farnesyl pyrophosphate; GUS: beta-glucuronidase; HMBC: heteronuclear multiple bond correlation; HMQC: heteronuclear multiple quantum coherence; HRT: *Hevea brasiliensis *rubber transferase; IPP: isopentenyl pyrophosphate; NMR: nuclear magnetic resonance; RER2: *Saccharomyces cerevisiae *dehydrodolichyl diphosphate synthase; SEC: size exclusion chromatography; SRT1: *Saccharomyces cerevisiae cis*-1,4-polyprenylcistransferases; TEM: transmission electron microscopy; TkCPT: *Taraxacum koksaghyz cis*-1,4-polyprenylcistransferases; TPT: *trans*-prenyltransferase

## Authors' contributions

TS contributed to results presented in figures 1, 3 and 4. ML generated antibodies and performed experiments for figures 5 and 7. AH, NvD and OM performed heterologous expression of TkCPT in tobacco protoplasts (Table 3) and yeast (Figure 7). RR did the transmission electron microscopy (Figure 1) and WE the nuclear magnetic resonance spectroscopy (Figures 2 and 3c). RF helped with the interpretation of data. DP and CSG set up the experimental outline and prepared the manuscript. All authors read and approved the final manuscript.

## Funding

This work was supported by a grant from the Ministry of Science and Education of Germany (grant no. FKZ 0313712), by EVONIK Industries AG, by the Hans-Fischer Gesellschaft Munich (to WE), by the Deutsche Bundesstiftung Umwelt (to ML) and the federal state North-Rhine Westphalia co-financed by the European Union.
